# Responses of plant biomass, photosynthesis and lipid peroxidation to warming and precipitation change in two dominant species (*Stipa grandis* and *Leymus chinensis*) from North China Grasslands

**DOI:** 10.1002/ece3.1982

**Published:** 2016-02-20

**Authors:** Xiliang Song, Yuhui Wang, Xiaomin Lv

**Affiliations:** ^1^ State Key Laboratory of Vegetation and Environmental ChangeInstitute of BotanyChinese Academy of ScienceBeijingChina

**Keywords:** Biomass, climate change, *Leymus chinensis*, lipid peroxidation, photosynthesis, precipitation change, *Stipa grandis*, warming

## Abstract

Influential factors of global change affect plant carbon uptake and biomass simultaneously. Although the effects from warming and precipitation change have been extensive studied separately, the responses of plant biomass, photosynthesis, and lipid peroxidation to the interaction of these factors are still not fully understood. In this study, we examined the physiological responses of two dominant plant species from grasslands of northern China with different functional traits to combinations of five simulated warming patterns and five simulated precipitation patterns in environment‐controlled chambers. Our results showed that the biomass, net CO_2_ assimilation rate (*P*_n_), maximal efficiency of photosystem II photochemistry (*F*_v_/*F*_m_), and chlorophyll content (Chl) of *Stipa grandis* and *Leymus chinensis* were enhanced by moderate warming and plus precipitation, but they declined drastically with high temperature and drought. High temperature and drought also led to significant malondialdehyde (MDA) accumulation, which had a negative correlation with leaf biomass. The lower level of lipid peroxidation in leaves of *S*.* grandis* suggests that this species is better protected from oxidative damage under heat stress, drought stress and their interactive conditions than *L*.* chinensis*. Using the subordinate function values method, we found *S*.* grandis* to be more sensitive to climate change than *L*.* chinensis* and the gross biomass and root biomass of *S*.* grandis* and the leaf biomass of *L*.* chinensis* were most sensitive to climate change. Furthermore, the *P*_n_ of both *S*.* grandis* and *L*.* chinensis* had a significant linear relationship with *F*_v_/*F*_m_ and Chl, indicating that carbon assimilation may be caused by nonstomatal limitations.

## Introduction

It is predicted that the global averaged surface temperature will increase by 1.5°C to 4.0°C and that extreme precipitation events will occur more frequently by the end of the 21st century (IPCC 2013). Rising temperature (especially temperature at high levels) caused a more severe water deficit by increasing potential evapotranspiration, aggravated ecosystem vulnerability, and exaggerated the aridification or desertification of arid areas (Maestre et al., 2012; Sivakumar 2007; Bai et al. 2004). Both temperature and water availability are important abiotic factors that determine plant productivity by affecting plant physiological processes and the structure and function of ecosystems, such as photosynthesis, growth, species composition, and geographical distribution (Erice et al. 2006; Albert et al. 2011; Dreesen et al. 2012; Xu et al. 2013b). It is necessary to understand the responses of plants to warming, precipitation change, and their interactions before investigating the adaptation and sensitivity of vegetation to future climate change.

Global climate warming, which has been proved by the increase in daily, seasonal, and annual mean temperature (Yamori et al. 2014), is expected to have a diverse and intense impact on plants, ecology, and biological systems (Martinez et al. 2014). In most cases, an increase in temperature below the optimum temperature is beneficial to plants. However, higher than expected temperature (heat stress) often produces negative effects on plant photosynthesis, growth, productivity, and water use efficiency (De Boeck et al. 2006; Campbell et al. 2007; Tang et al. 2007; Ainsworth and Ort 2010; Albert et al. 2011; Bauweraerts et al. 2013). For instance, Jin et al. (2011) found that moderate warming (+2.5°C) significantly increased the total weight of *Arabidopsis thaliana* seeds by approximately 37%. However, an increase of 5°C resulted in a reduction by approximately 14%, suggesting that lower levels of warming may be favorable for plants, whereas higher levels of warming may produce adverse results.

In global arid and semiarid areas, changing water conditions represents a dramatic impact on plant functions, such as growth and photosynthesis, which in turn affect global terrestrial ecosystem productivity (Bai et al. 2004; Chaves et al. 2009; Salazar‐Parra et al. 2015). Increased precipitation is expected to be favorable to plant photosynthesis and growth, species richness, plant community coverage (Wu et al. 2011; Yang et al. 2011). In contrast, drought caused by less precipitation has the opposite effect on plant growth, biomass, and ecosystem carbon flux (Wu et al. 2011; Farfan‐Vignolo and Asard 2012). According to the research of Issarakraisila et al. (2007), water stress reduces the fresh weight, leaf area, and dry weight of leafy vegetables by more than 50%. Furthermore, severe drought has detrimental effects on the photosynthetic apparatus, such as damage to chloroplasts (Xu et al. 2009), altered chlorophyll concentration (Ramírez et al. 2014), reduced photosynthetic enzyme activity (Chaves et al. 2003), and decreased efficiency of photosystem II (PSII) photochemistry (Xu and Zhou 2006).

Combined factors, such as water stress and high temperature, pose markedly higher constrains on plant growth and photosynthetic capacity than they do individually (Xu and Zhou 2006; Albert et al. 2011; Thomey et al. 2011; Bauweraerts et al. 2013). However, Wu et al. (2011) found that ecosystem responses to the combination of altered precipitation and warming tended to be weaker than the sum of the expected values of single‐factor responses. According to the results of controlled environment experiments, plant biomass could be enhanced by warming under normal precipitation, but it declines drastically with severe drought. For example, warming of 6°C increased the individual biomass of the C_4_ grass *Cleistogenes Suarrosa* under additional precipitation, whereas the reverse results were obtained under moderate and severe drought conditions (Xu et al. 2013a).

Lipid peroxidation refers to a series of free radicals reactions conducted in unsaturated fatty acids (Elstner 1982) and has been widely used as an indicator of cell oxidative damage (Foyer et al. 1994; Sofo et al. 2004; DaCosta and Huang 2007; Talbi et al. 2015). An increase in lipid peroxidation under prolonged stress indicates the declining scavenging ability within plant cells (Liu and Huang 2000), roots (Liang et al. 2003), and leaves (Sofo et al. 2004). Malondialdehyde (MDA), which reflects lipid peroxidation in plant cells and the responses to external stress (Prasad 1996; Thompson et al. 1998), is a major product of lipid peroxidation induced mainly by active oxygen species and a useful marker for oxidative stress (Cakmak and Horst 1991). Drought and heat stress increased MDA content of leaves (Reddy et al. 2004; Yang et al. 2014; Talbi et al. 2015), and their combination caused earlier and more severe oxidative damage to the leaf membrane integrity (Jiang and Huang 2001; Xu and Zhou 2005; Sekmen et al. 2014). The level of MDA was not only associated with drought and heat stress resistance but also has an adverse relationship with photosynthesis. Ali et al. (2005) results showed that heat stress (40°C) causes oxidative damage, which may play a primary role in the decrease in photochemical efficiency, and the leaves were found to be heavily affected with increased MDA levels. Xu also proved that the accumulation of MDA caused by soil water deficit and high temperature has an adverse impact on photosynthesis, indicating that peroxidation is closely associated with photosynthesis (Xu et al. 2009, 2011). Hence, antioxidant defense mechanisms are important for us to be able to estimate the plant's responses to future climate change especially environmental stress.

Grassland is one of the most widespread vegetation types in China, the total area of natural grassland in China which covers nearly 40% of China's land surface amounts to 392.8 million hectares (Chen and Fischer 1998). The grasslands dominated by *Stipa grandis* (a perennial bunchgrass) and *Leymus chinensis* (a native, clonal perennial rhizomatous grass) are two dominant vegetation types that widely distributed from the eastern Eurasian steppe to the middle Eurasian steppe zone, and they provide good livestock forage in Inner Mongolia (Wang and Gao 2003; Bai et al. 2008). The peak standing crop and peak aboveground live biomass were 152.12 g/m^2^ and 144.43 g/m^2^ for *S*.* grandis* steppe, 193.48 g/m^2^ and 182.68 g/m^2^ for *L*.* chinensis* steppe from 1980 to 1989, respectively (Xiao et al. 1995). Several experiments on *S*.* grandis* and *L*.* chinensis* under high temperature and water stress have been conducted by Xu and others (Xu et al. 2004, 2013a; Xu and Zhou 2005, 2006); they indicated that soil drought and high temperature destroy the function and integration of PSα and decrease plant biomass and high temperature weakens the adaptability of *L*.* chinensis* to drought. However, those studies mainly focused on single climate factor, the variations of plant biomass and potential photosynthetic capacity of *S*.* grandis* and *L*.* chin*ensis from north China grassland under warming coupled with precipitation change across a wide range remain unclear. Hence, the main objective of our research is to explore how future climate change affects the cell membrane peroxidation, photosynthetic characteristics and dry matter allocation of *S*.* grandis* and *L*.* chinensis*. In the present study, we hypothesized that (1) moderate warming and plus precipitation may enhance the biomass and photosynthetic capability of *S. grandis* and *L*.* chinensis*, where high temperature and drought may have the opposite effect. (2) There exists a significant interaction between warming and precipitation change, and their combined effect tends to be greater than that expected from the single‐factor responses.

## Materials and Methods

### Experimental site and setup

To address the combined effects of warming and precipitation change on *S*.* grandis* and *L*.* chinensis*'s biomass, photosynthesis, and lipid peroxidation characteristics, various water and heat conditions were controlled for seedlings germinated from seeds. The experiments were carried out at the experimental farm of the Institute of Botany, Chinese Academy of Science (39°48′N, 116°28′E, 67 m elevation above sea level), Beijing, China, from September to November in 2011. The seeds were obtained from grassland in Xilinhot, Inner Mongolia, China (41°43′N, 111°52′E, 1100 m elevation above sea level). The seeds of the two species were sterilized by 0.7% potassium permanganate solution for 8 min and then rinsed. They were sown in plastic pots (18 cm in diameter, 20 cm in height) wrapped with plastic film. Each plastic pot was filled with 4.08 kg of dry soil, which was obtained from a natural field grassland in the Xilinguole (Inner Mongolia, China),organic carbon content was 12.3 g/kg, and total nitrogen content was 1.45 g/kg. Polyethylene pots (10.9 cm in diameter, 9.5 cm in height) were used as the experimental containers, which were lined with plastic bags to prevent water leakage. In the present experiment, six replicates were used for each of the five water and five temperature treatments. Then, 150 pots with healthy plants were randomly selected and placed into five artificial control chambers (RXZ–500D, Ningbo Southeast Instrument Company, China) as different treatments.

**Figure 1:**
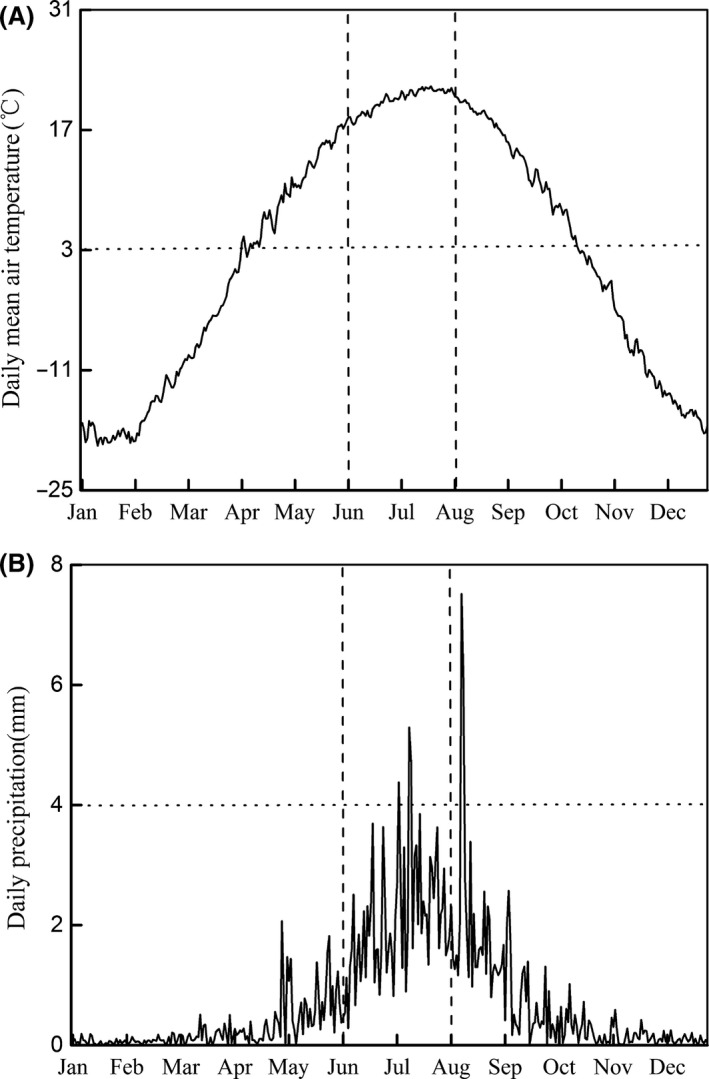
Monthly variation in mean air temperature (A) and precipitation (B) in Xilinhot during 1978–2007.

According to the monthly average temperature and precipitation during the *S*.* grandis* and *L*.* chinensis* during blooming season (June, July, and August) from 1978 to 2007 (see Fig. 1), five temperature and precipitation gradients were established. The five temperature gradients were (1) the current monthly average temperature (*T*_0_, the average temperature over 30 years); (2) *T*_0_ + 1.5°C (*T*_1.5_); (3) *T*_0_ + 2°C (*T*_2_); (4) *T*_0_ + 4°C (*T*_4_); and (5) *T*_0_ + 6°C (*T*_6_). The temperature treatments were controlled in artificial control chambers with different daytime and nighttime temperatures. The average temperatures of day and night for different months are listed in Table 1. Five different water gradients were set as follows: the current monthly precipitation (*W*_0_); W_0_ increased by 30% (*W*_+30_); W_0_ increased by 15% (*W*_+15_); W_0_ decreased by 15% (*W*_−15_); and W_0_ decreased by 30% (*W*_−30_). Quantitative irrigation was conducted every 3 days. The average monthly precipitation and the irrigation amount of each water treatment are listed in Table 2.

**Table 1:** Average daytime/nighttime temperatures and daily average temperature in months 6–8 during 1978–2007 (30 years)

Month	Temperature (°C)	
	Daytime/nighttime	Daily average temperature
June	22.1/16.1	19.2
July	24.4/18.4	22.8
August	22.6/16.6	19.6

**Table 2:** Average monthly precipitation of 6–8 during 1978–2007 (30 years) and the irrigation amount each time

Month	Precipitation (mm)	Irrigation amount (mL)				
		*W*_−30_	*W*_−15_	*W*_0_	*W*_+15_	*W*_+30_
June	44.9	26.5	32.2	37.9	43.6	49.3
July	77.9	46.0	55.9	65.8	75.6	85.5
August	64.5	38.1	46.3	54.4	62.6	70.8

### Biomass determinations

Biomass was measured 90 days after the plants had been moved into the environment‐control chambers using three pots for each treatment. During harvesting, the plants were divided into four parts: green leaves, dead leaves, roots, and stems for each species. The samples were dried at 80°C to constant weight and then weighed.

We used the sensitivity subordinate value (SV) of the ambiguity function as an evaluation index which can reflect the biomass sensitivity of different species. SV was estimated by equations (1) and (2):

(1) $$Y_{\mathrm{ij}}=1-\frac{X_{\mathrm{ij}}-X_{i\min}}{X_{i\max}-X_{i\min}}$$

(2) $$\text{SV}={\bar{Y}}_{i}=\frac{1}{n}\sum_{j=1}^{n}Y_{\mathrm{ij}}$$

where *X* denotes the plant biomass (g); *i*, different plant species; *j*, warming and precipitation change treatment; *X*_*i*min_, the minimum value for *i* in all treatments; *X*_*i*max_, the maximum value for *i* in all treatments; *Y*_ij_, the subordinate value for *i* in *j* treatment; ${\bar{Y}}_{i}$, the sensitivity subordinate value for *i*.

### Photosynthetic parameter measurements

Three plants from each treatment were chosen from different pots. Gas exchange parameter measurements were taken on healthy and youngest fully expanded leaves. Net photosynthetic rate per unit leaf area (*P*_n_) and stomatal conductance (*G*_s_) were measured using an open gas exchange system (LI‐6400, Li‐COR Inc., Lincoln, NE) linked to a leaf chamber fluorometer attachment (LI‐6400‐40, Li‐COR, Inc.) at the blooming stage of *S*.* grandis* and *L*.* chin*ensis, between 08:30 h and 11:30 h daily using red‐blue LED light as the illumination. Before taking measurements, the chosen leaves were acclimated in normal environmental conditions (with a saturated photosynthetic photon flux density of 900 *μ*mol/m^2^/sec, an ambient CO_2_ concentration of 390 *μ*mol/mol and temperature of 25°C) for 10 min.

The determination of chlorophyll fluorescence was conducted before predawn for complete dark adaptation using the same leaves that were used for gas exchange measurement. In order not to induce any significant photosynthetic reaction, the minimal fluorescence yield (*F*_0_) was measured using modulated light that was sufficiently low (<0.1 *μ*mol/m^2^/sec). The maximal fluorescence yield (*F*_m_) was measured by a 0.8 sec saturating pulse at 8000 *μ*mol/m^2^/sec on the already dark‐adapted leaves. Then, the maximal efficiency of PSII photochemistry was expressed as *F*_v_/*F*_m_ = (*F*_m_−*F*_0_)/*F*_m_ (Maxwell and Johnson 2000; Gorbe and Calatayud 2012).

### Determination of chlorophyll content

Samples were obtained from the leaves used for measurement of photosynthetic parameters, with three replicates per treatment. The procedure was carried out at 4°C in dark conditions by mashing a leaf sample (0.25 g) with a mortar and pestle with 80% acetone (v/v), filtering the extract through two layers of nylon centrifuging at 15,000 g in sealed tubes for 5 min, collecting, and then reading the supernatant at 663 and 647 nm to determine chlorophyll a and chlorophyll b, respectively. The equations of Lichtenthaler and Buschmann (2001) were used to determine the concentrations of chlorophyll a and chlorophyll b in mg/mL in the extract solution:

Chlorophyll a = 12.25 A_663_−2.79 A_647_,

Chlorophyll b = 21.50 A_647_−5.10 A_663_,

Chlorophyll content = Chlorophyll a + Chlorophyll b.

### Determination of malondialdehyde content

Leaves were obtained before the biomass was harvested, with three replicates per treatment. Fresh leaf material (1.00 g) from the leaf middle section was homogenized in 0.1% trichloroacetic acid (TCA) solution (2 mL, pH 7.0). The homogenate was centrifuged at 15,000 g for 10 min. To 1.5 mL of thiobarbituric acid (TBA) in 20% TCA, 0.5 mL of the supernatant was added. The mixture was incubated at 90°C in a shaking water bath for 20 min, and the reaction was stopped by placing the reaction tubes in an ice water bath (0°C). The samples were then centrifuged at 10,000 g for 5 min. The absorbance of the supernatant was read at 532 nm (Hernández and Almansa 2002), and the value for nonspecific absorption was subtracted at 600 nm. An absorption coefficient of 155 per mmol/L/cm was used to calculate the amount of MDA (Cakmak and Horst 1991).

### Statistical analysis

All experiments were conducted in a completely randomized block design with three replicates. All statistical analyses were performed using SPSS 18.0 (SPSS, Chicago, IL). The mean with standard error (±SE) is shown for each treatment, and the data normality of all parameters was tested before doing variance analysis. Effects of temperature or precipitation change on biomass, photosynthetic parameters, chlorophyll content, and MDA content were assessed by one‐/two‐way analysis of variance (ANOVA, *P *<* *0.05) followed by Duncan's multiple range test (Duncan, 1955). The graphing was performed using Origin 9.0 software (Origin Lab, Massachusetts, USA).

## Results

### Biomass accumulation

At the condition of *W*_0_, for *S*.* grandis*, the *T*_1.5_ and *T*_2_ treatments were beneficial to the indices of leaf biomass, stem biomass, root biomass, and gross biomass, but the *T*_4_ and *T*_6_ treatment significantly decreased these biomass indices. For *L*.* chinensis*, the *T*_1.5_, *T*_2_, and *T*_4_ treatments significantly increased all biomass indices, and the *T*_6_ treatment increased leaf biomass and stem biomass but decreased root biomass and gross biomass significantly (Table S1). Under different precipitation conditions, the biomass responses of *S*.* grandis* and *L*.* chinensis* to temperature change were different. Under lower precipitation conditions (*W*_−15_ and *W*_−30_), almost all the biomass indices of the two species decreased drastically with increasing temperature. Under higher precipitation levels (*W*_+15_ and *W*_+30_), all the biomass indices showed a trend of rising first and then falling with increasing temperature. From Table S1, we found that the biomass of *S*.* grandis* and *L*.* chinensis* had the highest values in the *T*_2_ × *W*_+30_ treatment and lowest values in the *T*_6_ × *W*_−30_ treatment. Compared to *T*_0_ × *W*_0_, the *T*_2_ × *W*_+30_ treatment increased leaf biomass, stem biomass, root biomass, and gross biomass of *S*.* grandis* and *L*.* chinensis* by 35.3% and 172.7%, 120.0% and 80.0%, 81.3% and 62.9%, and 74.3% and 80.4%, respectively. The *T*_6_ × *W*_−30_ treatment decreased the leaf biomass, stem biomass, root biomass, and gross biomass of *S*.* grandis* and *L*.* chinensis* by 41.2% and 27.3%, 60.0% and 60.0%, 47.9% and 68.6%, and 44.3% and 58.8%, respectively.

### Biomass sensitivity

Based on sensitivity subordinate values, we analyzed the biomass indices of *L*.* chinensis* and *S*.* grandis*, which are most sensitive to warming and precipitation change (Table S3). The results showed that the sensitivity subordinate values of *S*.* grandis* were leaf biomass (SV = 0.65), stem biomass (SV = 0.58), root biomass (SV = 0.68), and gross biomass (SV = 0.68), and the sensitivity subordinate values of *L*.* chinensis* were leaf biomass (SV = 0.62), stem biomass (SV = 0.45), root biomass (SV = 0.51), and gross biomass (SV = 0.52). These values indicate that the gross biomass and root biomass of *S*.* grandis* and the leaf biomass of *L*.* chinensis* are the most sensitive indices to warming and precipitation change and the higher value of SV for *S*.* grandis* than that of *L*.* chinensis* shows that *S*.* grandis* is more sensitive than *L*.* chinensis*.

### Chlorophyll content and lipid peroxidation

**Figure 2:**
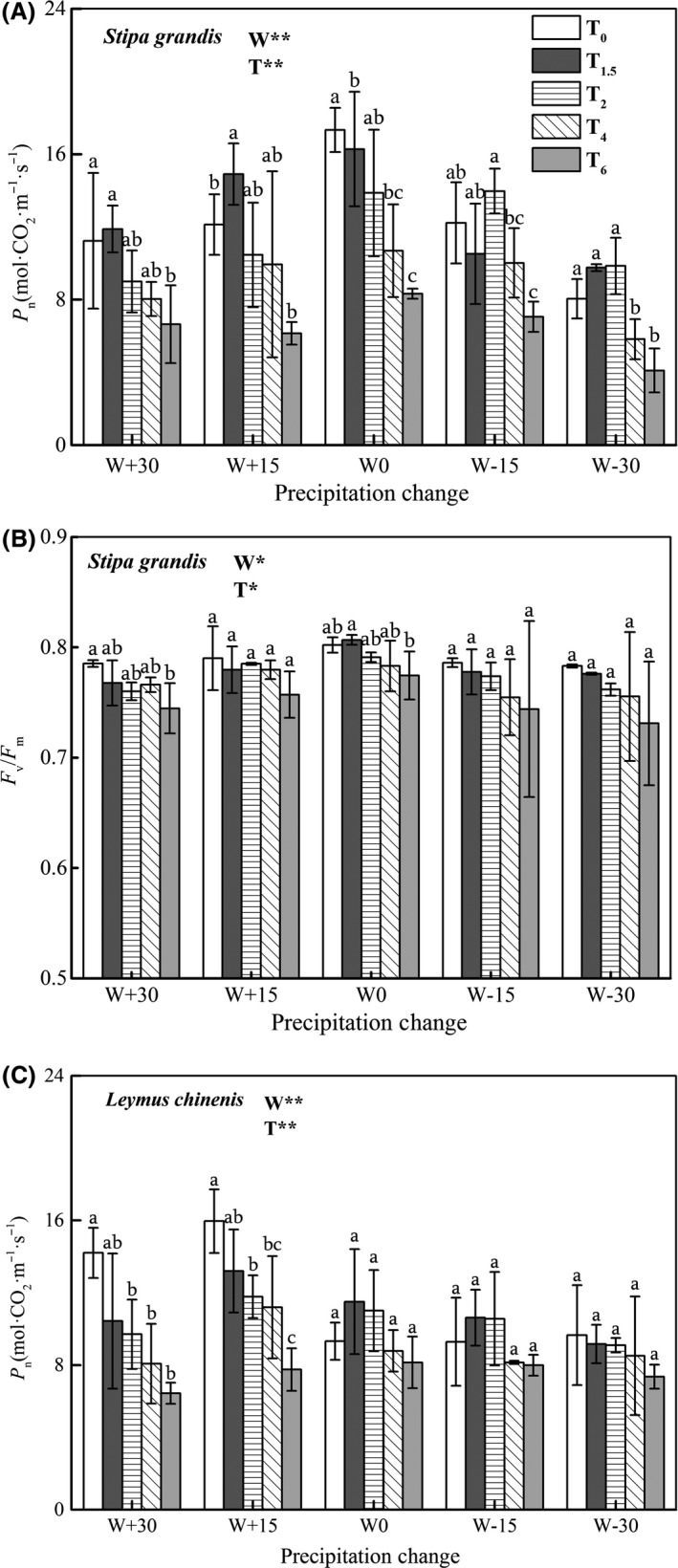
Combined effects of warming and watering on malondialdehyde contents of *Stipa grandis* and *Leymus chinensis*. T, temperature treatment; W, precipitation change treatment. Vertical bars represent ±SE of the mean (*n* = 3), and different letters on the SE bars indicate significant differences among the treatments (*P *<* *0.05). Significance levels are reported in figures as significant tendency with * when *P *<* *0.05 and ** when *P *<* *0.01.

Chl in *S*.* grandis* (Fig. 2A) and *L*. *chinensis* (Fig. 2B) was affected both by warming and precipitation change (*P *<* *0.01), but Chl was affected significantly (*P *<* *0.01) by the interaction of the two only in *L*. *chinensis*. For *S*.* grandis* and *L*. *chinensis*, Chl increased markedly (by 3.2–40.7% and 7.3–74.4%, respectively) at moderately elevated temperature but decreased markedly (by 8.7–30.9% and 3.6–65.4%, respectively) at high temperature compared with the control condition. Under warming conditions, there was no significant difference between precipitation treatments for *S*. *grandis*; but for *L*. *chinensis*, additional precipitation increased Chl and drought decreased it significantly.

**Figure 3:**
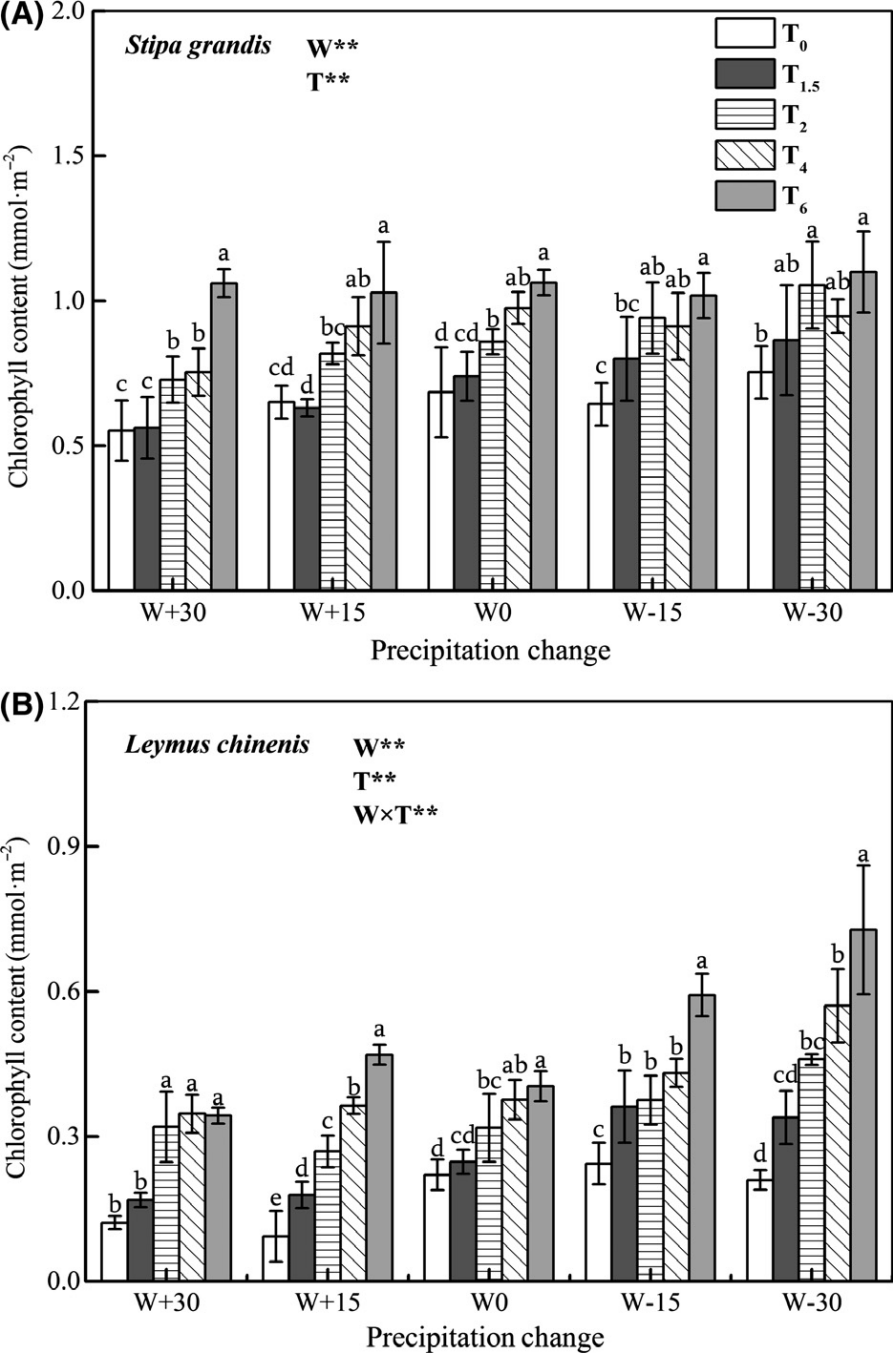
Combined effects of warming and watering on *P*_n_ and *F*_v_/*F*_m_ of *Stipa grandis* and *Leymus chinensis*. T, temperature treatment; W, precipitation change treatment. Vertical bars represent ±SE of the mean (*n* = 3); different letters on the SE bars indicate significant differences among the treatments (*P *<* *0.05). Significance levels are reported in figures as significant tendency with ** when *P *<* *0.01.

In all water conditions, compared to *T*_0_, the MDA content of *S*.* grandis* and *L*.* chinensis* was not enhanced by moderate warming (*T*_1.5_ and *T*_2_), but high temperature (*T*_4_ and *T*_6_) significantly increased the MDA content and the changes of *S*.* grandis* (Fig. 3A) and *L*.* chinensis* (Fig. 3B) were 22.6–82.3% and 37.8–129.5%, respectively. Under different warming conditions, compared to *W*_0_, decreased precipitation (*W*_−15_ and *W*_−30_) significantly increased the MDA content of *S*.* grandis* and *L*.* chinensis* (except for *T*_4_ in *Stipa grandis* and *T*_1.5_ in *Leymus chinensis*) by 5.3–28.5% and 0.9–49.9%, where additional precipitation (*W*_+15_ and *W*_+30_) decreased the MDA content by 7.2–24.7% and 4.5–31.5%, respectively. Although the MDA content in both *S*. *grandis* and *L*. *chinensis* was affected by warming and precipitation change separately, only the MDA content of *L*.* chinensis* was affected significantly (*P *<* *0.01) by the interaction of the two.

### Photosynthetic capacity

**Figure 4:**
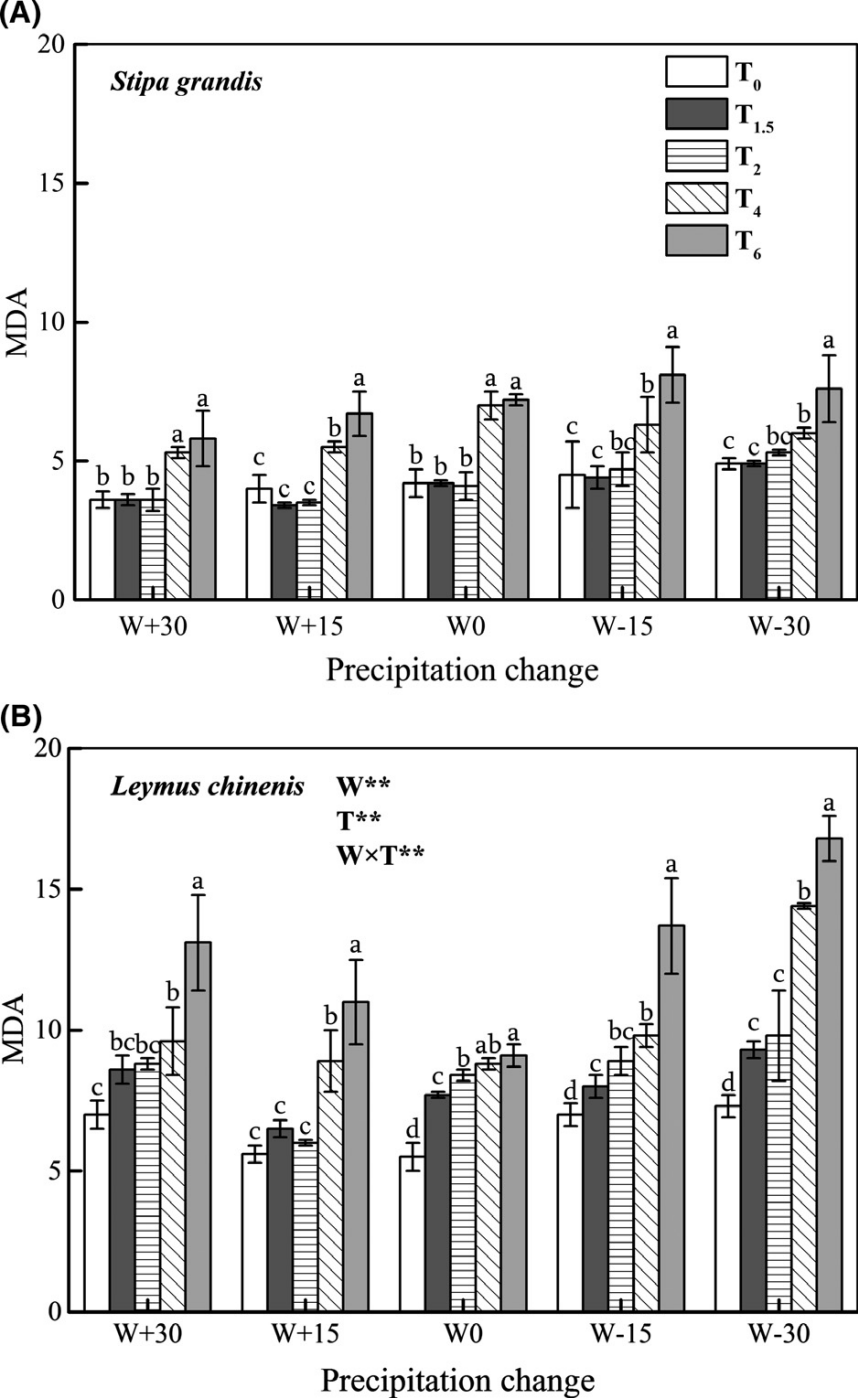
Combined effects of warming and watering on chlorophyll content of *Stipa grandis* and *Leymus chinensis*. T, temperature treatment; W, precipitation change treatment. Vertical bars represent ±SE of the mean (*n* = 3), and different letters on the SE bars indicate significant differences among the treatments (*P *<* *0.05). Significance levels are reported in figures as significant tendency with ** when *P *<* *0.01.

Figure 4A and C demonstrates that in all precipitation treatments, the *T*_1.5_ and *T*_2_ treatment caused no significant change (*P *>* *0.05) to the *P*_n_ of *S*.* grandis*, indicating that photosynthesis was not affected under moderate warming conditions. However, the *P*_n_ of *S*.* grandis* decreased drastically by 18.0–50.2% after warming in the *T*_4_ and *T*_6_ treatments, indicating that photosynthesis was inhibited by heat stress. For *L*.* chinensis*, there was no drastic changing on *P*_n_ (*P *>* *0.05) with temperature increase under the *W*_0_, *W*_−15_ and *W*_−30_ conditions, but *P*_n_ decreased by 17.3–54.6% under the *W*_+15_ and *W*_+30_ conditions. At the condition of *T*_0_, compared to *W*_0_, additional precipitation is favorable to the *P*_n_ of *S*. *grandis* and *L*. *chinensis*, whereas precipitation deficiency will produce a negative effect. Across all temperature and water treatments, *P*_n_ reached its the maximum value (9.8–16.6 mol CO_2_/m/sec and 9.2–13.2 mol CO_2_/m/sec,respectively) in the *T*_2_ × *W*_+15_ treatment and its lowest value (4.1–8.3 mol CO_2_/m/sec and 7.4–8.1 mol CO_2_/m/sec, respectively) in the *T*_6_ × *W*_−30_ treatment. Although the *P*_n_ of both *S*.* grandis* and *L*.* chinensis* was significantly affected (*P *<* *0.01) by warming and precipitation change, there was no significant interaction (*P *>* *0.05) between the two factors.

Both temperature increase and precipitation change had a significant effect (*P *<* *0.05) on *F*_v_/*F*_m_ of *S. grandis* and *L. chinensis* (Fig. 4B and D).Under all precipitation treatments, compared to ambient temperature (*T*_0_), the value of *F*_v_/*F*_m_ of *S. grandis* showed a slight decrease by 0.6–6.6% with temperature increase (except for *W*_0_ × *T*_1.5_) (Fig. 4B). Under *T*_0_, *T*_1.5_, and *T*_2_ conditions, compared to *W*_0_, the water treatment had no significant effect on *F*_v_/*F*_m_ (*P* > 0.05). However, under *T*_4_ and *T*_6_ condition, compared to *W*_0_, the water treatment decreased *F*_v_/*F*_m_ by 0.4–5.6%. For *L. chinensis*, under all precipitation treatments, compared to *T*_0_, *T*_1.5_ treatment increased *F*_v_/*F*_m_ by 0.1–1.1%, but the warming of 2°C to 6°C decreased *F*_v_/F_m_ by 0.3–6.5% (Fig. 4D). Under conditions *T*_0_ and *T*_1.5_ compared to *W*_0_, the water treatment had no significant effect on *F*_v_/*F*_m_ (*P* > 0.05). However, under *T*_2_, *T*_4_ and, *T*_6_ condition, compared to *W*_0_, the water treatment decreased *F*_v_/*F*_m_ by 0.5–4.6%.

**Figure 5:**
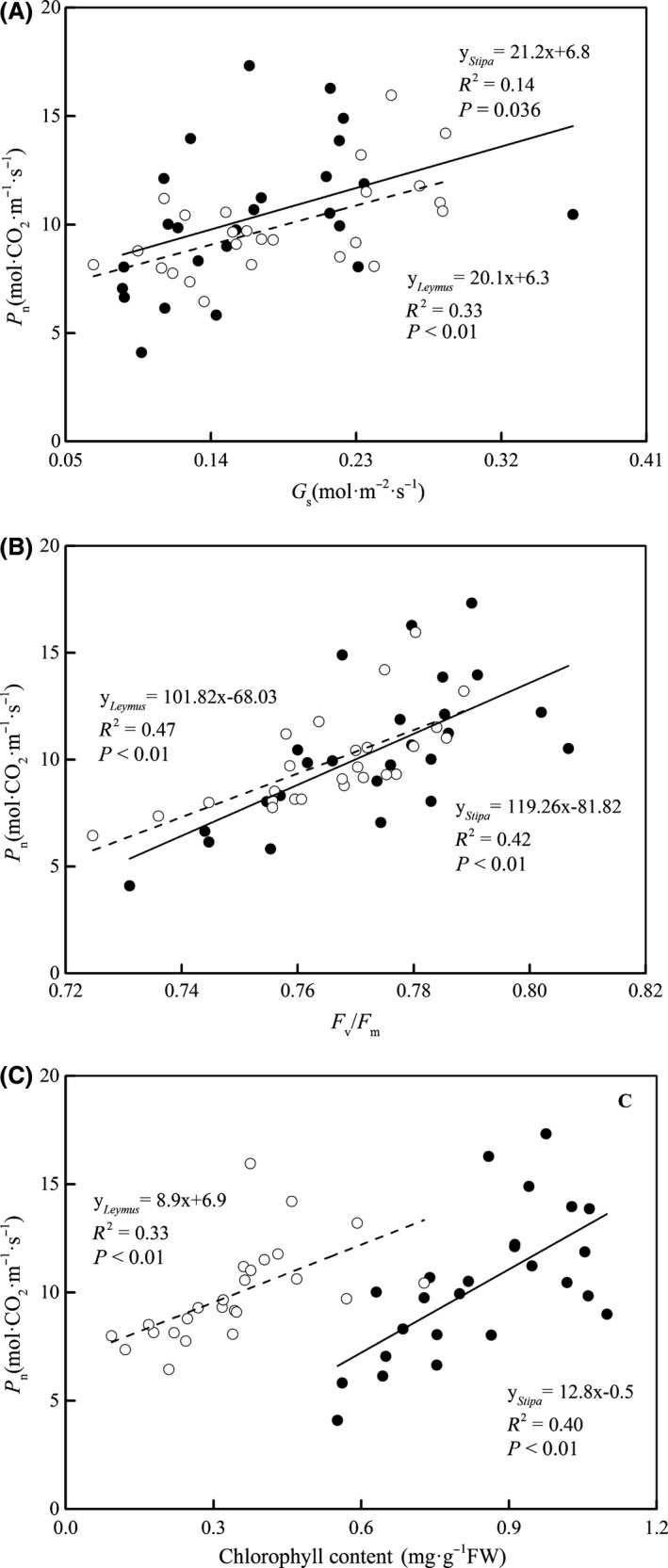
Linear correlation between net CO_2_ assimilation rate (*P*_n_, mol/m^2^/sec) and (A) stomatal conductance (Gs, mol/m^2^/sec, data not shown), (B) maximal efficiency of PSII photochemistry (*F*_v_/*F*_m_), and (C) chlorophyll content (mg/g FW).

Here, the relationships of *P*_n_ with *G*_s_, *F*_v_/*F*_m_ and Chl were demonstrated in Figure 5A–C. *P*_n_ was positively and significantly correlated with *F*_v_/*F*_m_ (*R* ^2^ = 0.42 in *S*.* grandis*;* R* ^2^ = 0.47 in *L*.* chinensis*) and Chl (*R* ^2^ = 0.40 in *S*.* grandis*;* R* ^2^ = 0.33 in *L*.* chinensis*) except for *G*_s_ (*R* ^2^ = 0.14 on *S*.* grandis*;* R* ^2^ = 0.33 on *L*.* chinensis*), indicating that stomatal conductance accounted for 14% and 33% of the change in *P*_n_, 42% and 47% for *F*_v_/*F*_m_ and 40% and 33% for Chl, respectively.

## Discussion

### Response and sensitivity of biomass allocation

Global warming and changing precipitation patterns caused by increasing CO_2_ concentration in the earth's atmosphere (IPCC, 2012) might result in irreversible changes in plant growth, biomass, and photosynthesis (Xu et al. 2013b). Generally, an increase in temperature will favor photosynthesis in leaves and plant growth. Norton et al. (1999) found that an increase of 3°C in air temperature significantly promoted the growth of *Agrostis curtisii*. However, higher temperature (+4°C) significantly decreased biomass and seed weights harvested from *Chenopodium Album* by 47.3% and 14.6%, respectively. Our study also found that moderate temperature increase (1.5°C to 2°C) is beneficial to the biomass accumulation of *S*.* grandis* and *L*.* chinensis*, but high temperature (4°C to 6°C increase) has negative effects. Many studies have indicated that additional precipitation could enhance plant growth and photosynthesis (Wu et al. 2011; Yang et al. 2011), and the water deficit has opposite effects (Issarakraisila et al. 2007; Wu et al. 2011). Our study yielded similar results. Additional precipitation significantly enhanced biomass accumulation of *S*.* grandis* and *L*.* chinensis*, whereas drought caused by decreased precipitation led to dramatic reductions. Furthermore, by analysis of variance, all the biomass indices of *S*.* grandis* and *L*.* chinensis* were significantly affected by warming and precipitation change, which exhibited a significant interaction (*P *<* *0.01, Table S2). The maximum biomass was found in the *T*_2_ × *W*_+30_ treatment and the minimum in the *T*_6_ × *W*_−30_ treatment, which indicates that a combination of warming and precipitation has a larger influence than does the sum of the single factors.

### The response mechanism of photosynthesis

Our research showed that the effects of different temperature and water treatments on the *P*_n_ of *S*.* grandis* and *L*.* chinensis* were not all the same. Moderate warming had little effect on *P*_n_, high temperature and drought treatment caused a significant decrease in *P*_n_, and additional precipitation treatment significantly increased *P*_n_ in both *S*.* grandis* and *L*.* chinensis*. Although both the warming and precipitation change treatment had a highly significant impact on the *P*_n_ of *S*.* grandis* and *L*.* chinensis*, there is always a contradictory theory regarding the influence of stomatal limitation and nonstomatal limitation on *P*_n_ under environmental stresses. For instance, Da Silva and Arrabaça (2004) showed evidence of the reduction in photosynthesis of a water‐stressed C_4_ grass is mainly attributed to stomatal limitation, while von Caemmerer et al. (2004) reported that stomatal limitation does not play a major role in photosynthesis change in transgenic tobacco plants.

Leaf chlorophyll content and the photochemical efficiency indicated by *F*_v_/*F*_m_ are related closely to the integrity of chloroplasts and are proportional to photosynthetic capacity (Krause and Weis 1991; Yoo et al. 2003). Chlorophyll content (Chl) is an important physiological indicator of a plant's photosynthtic potential, playing an important role in plant photosynthesis rate (Mae 1997; Lichtenthaler and Buschmann 2001; Jakob et al. 2005), primary biological productivity, and biomass (Jakob et al. 2005). It is also an effective indicator of environmental stress (Datt 1999). *F*_v_/*F*_m_ is the maximum photochemical efficiency of PSII under dark adaptation. Having been widely used for the detection of photoinhibition, it reflects the maximum efficiency of the photosynthetic apparatus that converts absorbed light energy into chemical reactions (Dickmann et al., 1992; Herppich and Peckmann 2000). The present results (Fig. 5) showed that *P*_n_ had significant and positive relationships with *F*_v_/*F*_m_ and Chl (*P *<* *0.05), but had no significant relationship with *G*_s_, indicating photosynthetic capacity may be depressed by Chl and PSII activity under warming and precipitation change conditions. Thus, nonstomatal limitation may play a major role in determining the carbon assimilation rate in our experimental conditions. This result could also be seen in the research of Xu et al. (2011).

### Response of lipid peroxidation damage

**Figure 6:**
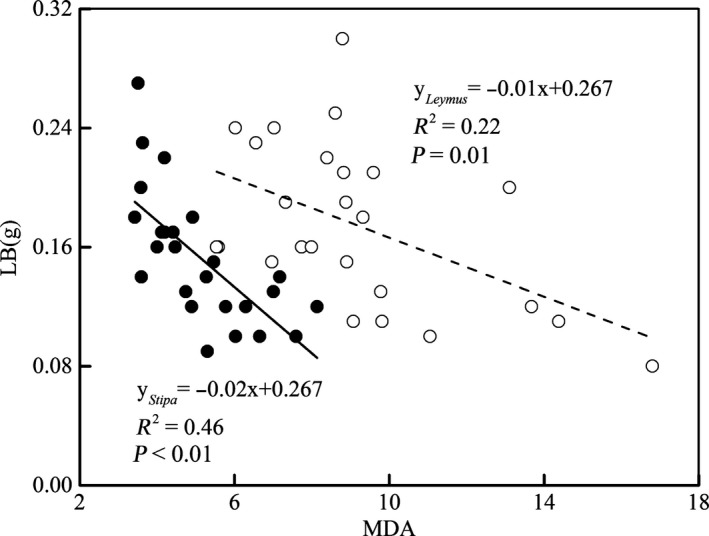
Linear correlation between leaf biomass and MDA content.

The importance of antioxidant defenses has been identified in a number of studies, and they have been widely reported to play a key role in environmental stress (Farrant et al. 2004; Ogweno et al. 2008; Xu et al. 2011). Malondialdehyde (MDA) is a product of peroxidation of unsaturated fatty acids in phospholipids and is responsible for cell membrane damage. The higher of MDA content, the larger of cell membrane lipid peroxidation will be damaged. The MDA contents of leaves were found to be significantly higher in *Agrostis stolonifera* after heat stress treatment for 14, 28, 42, and 56 days (Liu and Huang 2000). Such increases have also been found in other species under drought (Zhang and Kirkham 1994), which means great damages are caused to the structure and function of the cell membrane by heat stress and drought. In the present study, compared with the present weather conditions, the MDA content of leaf segments of *S*.* grandis* and *L*.* chinensis* showed no obvious change under moderate warming and higher precipitation conditions, but it significantly increased under high temperature (warming 4°C to 6°C) and drought stress (precipitation decrease 15–30%). An enhanced level of lipid peroxidation was observed in leaf segments of *S*.* grandis* and *L*.* chinensis* under high temperature and drought stress, which is similar to a study on maize (Xu et al. 2011) and *L*. *chinensis* (Xu and Zhou 2006; Xu et al. 2009). This finding may be due to the generation of active oxygen species (AOS) under temperature stress and drought as reported by other authors (Moran et al. 1994; Ali et al. 2005; DaCosta and Huang 2007). Xu found that the lipid peroxidation is closely associated with photosynthesis and that biomass accumulation is limited by photosynthesis (Xu et al. 2009, 2011), so we speculate that lipid peroxidation may have a certain relationship with the biomass of *S*.* grandis* and *L*.* chinensis*. Figure 6 illustrates that the leaf biomass of *S*.* grandis* and *L*.* chinensis* has a significantly linear negative relationship with MDA content (*P* ≦ 0.01), indicating that one reason for the decrease in leaf biomass caused by heat stress and drought is damage from lipid peroxidation. Furthermore, the lower level of lipid peroxidation in leaves of *S*.* grandis* suggests that this cultivar is better protected from oxidative damage under heat stress, drought stress, and their interactive conditions than *L*.* chinensis*.

## Conflict of Interest

None declared.

## Supporting information


**Table S1.** Combined effects of warming and watering on plant biomass of *Stipa grandis* and *Leymus chinenis*.
**Table S2.** Variance analysis of *biomass* of *Stipa grandis* and *Leymus chinenis* between different warming and precipitation treatments.
**Table S3.** The biomass sensitivity subordinate values (SV) of *Stipa grandis* and *Leymus chinenis* between different warming and precipitation treatments.
