# Supplementary material for: Responses of plant biomass, photosynthesis and lipid peroxidation to warming and precipitation change in two dominant species (Stipa grandis and Leymus chinensis) from North China Grasslands
Source: Ecol Evol. 2016 Feb 20;6(6):1871–82. doi: 10.1002/ece3.1982 (PMC4760990; doi:10.1002/ece3.1982)
Supplement: Supplementary file 1 — Table S1. Combined effects of warming and watering on plant biomass of Stipa grandis and Leymus chinenis. Table S2. Variance analysis of biomass of Stipa grandis and Leymus chinenis between different warming and precipitation treatments. Table S3. The biomass sensitivity subordinate values (SV) of Stipa grandis and Leymus chinenis between different warming and precipitation treatments. [file ECE3-6-1871-s001.docx]

**Table 1** Combined effects of warming and watering on plant biomass of *Stipa grandis* and *Leymus chinenis*

|  |  | *Stipa grandis* | | | |  | *Leymus chinenis* | | | |
| --- | --- | --- | --- | --- | --- | --- | --- | --- | --- | --- |
|  |  | LB(g) | SB(g) | RB(g) | GB(g) |  | LB(g) | SB(g) | RB(g) | GB(g) |
| W_-30_ | T_0_ | 0.18±0.03 a | 0.04±0.03 ab | 0.27±0.01 b | 0.49±0.07 ab |  | 0.19±0.02 a | 0.05±0.01 a | 0.56±0.02 a | 0.80±0.04 a |
|  | T_1.5_ | 0.12±0.02 b | 0.06±0.01 a | 0.43±0.07 a | 0.62±0.02 a |  | 0.18±0.02 a | 0.05±0.01 a | 0.36±0.02 b | 0.59±0.01 b |
|  | T_2_ | 0.09±0.02 b | 0.04±0.01 ab | 0.29±0.02 b | 0.42±0.14 b |  | 0.11±0.01 b | 0.05±0.01 a | 0.37±0.01 b | 0.54±0.02 c |
|  | T_4_ | 0.10±0.02b | 0.03±0.00 b | 0.36±0.02 ab | 0.49±0.01 ab |  | 0.11±0.02 b | 0.02±0.02 b | 0.31±0.02 c | 0.44±0.01 d |
|  | T_6_ | 0.10±0.03 b | 0.02±0.01 ab | 0.25±0.06 b | 0.39±0.02 b |  | 0.08±0.02 c | 0.02±0.0a b | 0.11±0.02 d | 0.21±0.03 e |
|  |  |  |  |  |  |  |  |  |  |  |
| W_-15_ | T_0_ | 0.16±0.02 ab | 0.06±0.01 a | 0.43±0.02a | 0.65±0.16 a |  | 0.24±0.01 a | 0.06±0.01 a | 0.58±0.02 a | 0.87±0.03 a |
|  | T_1.5_ | 0.17±0.02 a | 0.07±0.01 a | 0.41±0.07 a | 0.64±0.03 a |  | 0.16±0.01 b | 0.03±0.01 ab | 0.44±0.02 b | 0.63±0.02 b |
|  | T_2_ | 0.13±0.02 ab | 0.04±0.01 b | 0.39±0.05 a | 0.56±0.18 ab |  | 0.15±0.01 c | 0.05±0.01 ab | 0.40±0.01 c | 0.61±0.03 b |
|  | T_4_ | 0.12±0.03 b | 0.04±0.01 b | 0.36±0.05 a | 0.52±0.04 b |  | 0.13±0.01 c | 0.04±0.02 ab | 0.32±0.01 d | 0.49±0.04 c |
|  | T_6_ | 0.12±0.02 b | 0.03±0.01 b | 0.26±0.02 b | 0.41±0.13 c |  | 0.12±0.02 c | 0.03±0.02 b | 0.23±0.03 e | 0.38±0.01 d |
|  |  |  |  |  |  |  |  |  |  |  |
| W_0_ | T_0_ | 0.17±0.02 ab | 0.05±0.01 b | 0.48±0.07 ab | 0.70±0.06 b |  | 0.16±0.01 b | 0.05±0.04 bc | 0.35±0.03 c | 0.56±0.02 c |
|  | T_1.5_ | 0.22±0.04 a | 0.06±0.06 b | 0.51±0.03 ab | 0.79±0.06 a |  | 0.16±0.01 bc | 0.04±0.01 c | 0.42±0.02 b | 0.62±0.03 b |
|  | T_2_ | 0.17±0.04 ab | 0.10±0.02 a | 0.54±0.04 a | 0.81±0.02 a |  | 0.22±0.03 a | 0.08±0.01 a | 0.46±0.02 b | 0.77±0.03 b |
|  | T_4_ | 0.13±0.01 b | 0.06±0.01 b | 0.46±0.05 b | 0.65±0.04 b |  | 0.21±0.02 a | 0.08±0.01 a | 0.60±0.01 a | 0.90±0.02 a |
|  | T_6_ | 0.14±0.04 b | 0.04±0.03 b | 0.25±0.01 c | 0.43±0.05 c |  | 0.11±0.04 c | 0.07±0.02 ab | 0.25±0.05 d | 0.44±0.08 d |
|  |  |  |  |  |  |  |  |  |  |  |
| W_+15_ | T_0_ | 0.16±0.02 b | 0.05±0.01 b | 0.52±0.02 ab | 0.73±0.03 b |  | 0.16±0.01 c | 0.07±0.03 a | 0.35±0.01 c | 0.57±0.02 c |
|  | T_1.5_ | 0.18±0.02 b | 0.06±0.02 b | 0.61±0.02 a | 0.85±0.03 ab |  | 0.23±0.02 ab | 0.05±0.02 a | 0.52±0.02 b | 0.80±0.02 b |
|  | T_2_ | 0.27±0.04 a | 0.09±0.01 a | 0.60±0.03 a | 0.95±0.02 a |  | 0.24±0.04 a | 0.08±0.04 a | 0.61±0.01 a | 0.92±0.09 a |
|  | T_4_ | 0.15±0.01 b | 0.03±0.02 bc | 0.46±0.19 ab | 0.65±0.21 bc |  | 0.19±0.01 bc | 0.05±0.02 a | 0.51±0.02 b | 0.75±0.01 b |
|  | T_6_ | 0.10±0.01 c | 0.02±0.01 c | 0.38±0.01 b | 0.49±0.12 c |  | 0.10±0.03 d | 0.07±0.02 a | 0.27±0.04 d | 0.45±0.03 d |
|  |  |  |  |  |  |  |  |  |  |  |
| W_+30_ | T_0_ | 0.14±0.02 b | 0.10±0.03 a | 0.58±0.06 bc | 0.81±0.06 c |  | 0.15±0.03 d | 0.08±0.02 ab | 0.43±0.03 c | 0.65±0.01 d |
|  | T_1.5_ | 0.20±0.01 a | 0.11±0.01 a | 0.68±0.12 b | 0.99±0.12 b |  | 0.25±0.04 ab | 0.09±0.01 a | 0.57±0.05 b | 0.92±0.06 b |
|  | T_2_ | 0.23±0.05 a | 0.11±0.03 a | 0.87±0.08 a | 1.22±0.08 a |  | 0.30±0.02 a | 0.09±0.03 a | 0.75±0.03 a | 1.14±0.02 a |
|  | T_4_ | 0.14±0.03 b | 0.05±0.02 b | 0.46±0.01 cd | 0.64±0.03 d |  | 0.21±0.02 bc | 0.06±0.01 ab | 0.55±0.06 b | 0.82±0.08 c |
|  | T_6_ | 0.12±0.01 b | 0.03±0.01ba | 0.36±0.07 e | 0.51±0.08 d |  | 0.20±0.03 c | 0.06±0.01 b | 0.28±0.04 d | 0.53±0.02e |

Different lowercases indicate significant difference between different temperature treatments within the same precipitation treatment compared with control(*p*<0.05)

**Table. 2** Variance analysis of *biomass* of *Stipa grandis* and *Leymus chinenis* between different warming and precipitation treatments

|  | *Stipa grandis* | | | | | | | | | | |  | *Leymus chinenis* | | | | | | | | | | |
| --- | --- | --- | --- | --- | --- | --- | --- | --- | --- | --- | --- | --- | --- | --- | --- | --- | --- | --- | --- | --- | --- | --- | --- |
|  | T | | |  | W | | |  | T×W | | |  | T | | |  | W | | |  | T×W | | |
|  | df | F | P |  | df | F | P |  | df | F | P |  | df | F | P |  | df | F | P |  | df | F | P |
| LB | 4 | 20.904 | 0.000 |  | 4 | 13.158 | 0.000 |  | 16 | 6.138 | 0.000 |  | 4 | 59.792 | 0.000 |  | 4 | 35.429 | 0.000 |  | 16 | 6.696 | 0.000 |
| SB | 4 | 22.162 | 0.000 |  | 4 | 13.008 | 0.000 |  | 16 | 3.450 | 0.000 |  | 4 | 4.630 | 0.003 |  | 4 | 14.461 | 0.000 |  | 16 | 2.240 | 0.015 |
| RB | 4 | 29.811 | 0.000 |  | 4 | 36.390 | 0.000 |  | 16 | 4.032 | 0.000 |  | 4 | 260.474 | 0.000 |  | 4 | 86.117 | 0.000 |  | 16 | 46.834 | 0.000 |
| GB | 4 | 54.404 | 0.000 |  | 4 | 51.318 | 0.000 |  | 16 | 6.317 | 0.003 |  | 4 | 411.935 | 0.000 |  | 4 | 137.025 | 0.000 |  | 16 | 5.531 | 0.000 |

**Table. 3** The biomass sensitivity subordinate values(SV) of *Stipa grandis* and *Leymus chinenis* between different warming and precipitation treatments

|  |  | W_-30_ | | | | |  | W_-15_ | | | | |  | W_0_ | | | | |
| --- | --- | --- | --- | --- | --- | --- | --- | --- | --- | --- | --- | --- | --- | --- | --- | --- | --- | --- |
|  |  | T_0_ | T_1.5_ | T_2_ | T_4_ | T_6_ |  | T_0_ | T_1.5_ | T_2_ | T_4_ | T_6_ |  | T_0_ | T_1.5_ | T_2_ | T_4_ | T_6_ |
| *Stipa grandis* | LB | 0.49 | 0.83 | 1.00 | 0.96 | 0.92 |  | 0.62 | 0.57 | 0.79 | 0.81 | 0.85 |  | 0.55 | 0.26 | 0.53 | 0.75 | 0.72 |
|  | SB | 0.71 | 0.51 | 0.75 | 0.85 | 0.81 |  | 0.51 | 0.47 | 0.75 | 0.78 | 0.81 |  | 0.64 | 0.51 | 0.14 | 0.51 | 0.75 |
|  | RB | 0.98 | 0.71 | 0.94 | 0.82 | 1.00 |  | 0.71 | 0.75 | 0.77 | 0.82 | 1.00 |  | 0.64 | 0.59 | 0.54 | 0.67 | 1.00 |
|  | GB | 0.88 | 0.73 | 0.96 | 0.88 | 1.00 |  | 0.68 | 0.69 | 0.80 | 0.84 | 0.98 |  | 0.63 | 0.51 | 0.49 | 0.68 | 0.95 |
|  |  |  |  |  |  |  |  |  |  |  |  |  |  |  |  |  |  |  |
| *Leymus chinenis* | LB | 0.55 | 0.30 | 0.70 | 0.70 | 0.75 |  | 0.60 | 0.70 | 0.70 | 0.35 | 0.25 |  | 0.95 | 0.75 | 0.40 | 0.30 | 0.00 |
|  | SB | 0.52 | 0.52 | 0.52 | 0.95 | 1.00 |  | 0.71 | 0.48 | 0.81 | 0.52 | 0.86 |  | 0.52 | 0.00 | 0.67 | 0.10 | 0.19 |
|  | RB | 0.69 | 0.30 | 0.60 | 0.59 | 1.00 |  | 0.67 | 0.27 | 0.48 | 0.54 | 0.81 |  | 0.63 | 0.00 | 0.45 | 0.51 | 0.78 |
|  | GB | 0.37 | 0.29 | 0.62 | 0.61 | 0.53 |  | 0.59 | 0.55 | 0.56 | 0.37 | 0.24 |  | 0.65 | 0.57 | 0.40 | 0.24 | 0.00 |

|  |  |  | W_+15_ | | | | |  | W_+30_ | | | | |  | SV |
| --- | --- | --- | --- | --- | --- | --- | --- | --- | --- | --- | --- | --- | --- | --- | --- |
|  |  |  | T_0_ | T_1.5_ | T_2_ | T_4_ | T_6_ |  | T_0_ | T_1.5_ | T_2_ | T_4_ | T_6_ |  |  |
| *Stipa grandis* | LB |  | 0.58 | 0.49 | 0.00 | 0.66 | 0.97 |  | 0.74 | 0.38 | 0.19 | 0.72 | 0.83 |  | 0.65 |
|  | SB |  | 0.64 | 0.54 | 0.27 | 0.81 | 1.00 |  | 0.17 | 0.03 | 0.00 | 0.68 | 0.85 |  | 0.58 |
|  | RB |  | 0.57 | 0.42 | 0.44 | 0.66 | 0.80 |  | 0.47 | 0.31 | 0.00 | 0.67 | 0.83 |  | 0.68 |
|  | GB |  | 0.59 | 0.45 | 0.32 | 0.69 | 0.88 |  | 0.49 | 0.27 | 0.00 | 0.69 | 0.86 |  | 0.68 |
|  |  |  |  |  |  |  |  |  |  |  |  |  |  |  |  |
| *Leymus chinenis* | LB |  | 0.55 | 0.85 | 0.45 | 0.55 | 0.45 |  | 1.00 | 0.90 | 0.95 | 1.00 | 0.50 |  | 0.62 |
|  | SB |  | 0.36 | 0.19 | 0.52 | 0.52 | 0.24 |  | 0.19 | 0.10 | 0.00 | 0.38 | 0.48 |  | 0.45 |
|  | RB |  | 0.62 | 0.22 | 0.37 | 0.36 | 0.74 |  | 0.51 | 0.23 | 0.28 | 0.31 | 0.74 |  | 0.51 |
|  | GB |  | 0.75 | 0.70 | 0.26 | 0.42 | 0.34 |  | 1.00 | 0.82 | 0.75 | 0.74 | 0.66 |  | 0.52 |
